# Determinants of Patient Satisfaction With Primary Health Care Among the Rural Community People: A Cross‐Sectional Evidence From Khulna Division, Bangladesh

**DOI:** 10.1002/hsr2.73043

**Published:** 2026-08-18

**Authors:** Md. Moshiuzzaman Adnan, Afsana Tanni, Israt Jahan, Suvasish Das Shuvo

**Affiliations:** ^1^ Department of Nutrition and Food Technology Jashore University of Science and Technology Jashore Bangladesh; ^2^ School of Medical, Indigenous, and Health Sciences University of Wollongong Wollongong NSW Australia

**Keywords:** chronic disease, patient satisfaction, primary health care, rural community people, utilization

## Abstract

**Background and Aims:**

The primary healthcare system in Bangladesh is significantly inadequate in terms of quality and accessibility, particularly in rural areas. Patient satisfaction (PS) has been associated with utilization of primary health care (PHC) facilities. This study aimed to determine the factors associated with patient satisfaction in primary health care facilities among the rural community people in Khulna Division, Bangladesh.

**Methods:**

A cross‐sectional survey was conducted among 721 adults in three districts of the Khulna division including Jashore, Khulna, and Kushtia. PS was measured using five‐point Likert scale (7 items, total 0‐28 score). Data was gathered through a structured questionnaire and analyzed utilizing SPSS software and R programming.

**Results:**

The overall patient satisfaction score was 18.41 ± 3.49 out of 28, representing 65.8%. Sociodemographic characteristics such as participants aged 41–50 years (*β* = −0.69; *p* = 0.034), having chronic diseases (*β* = −0.77; *p* = 0.047) and health facilities like union health and family welfare center (UHFWC) (*β* = −1.7; *p* = 0.028) were significantly associated with lower PS score. Regarding utilization factors, quality of treatment (*β* = 1.5; *p* = 0.036) and feeling comfortable to ask health provider (*β* = 2.8; *p* = 0.038) were significantly associated with higher PS score while waiting time more than 30 min (*β* = −1.7; *p* = 0.023) and getting free medicine (*β* = −1.7; *p* = 0.032) were significant predictors of lowering PS score.

**Conclusion:**

The Overall PS among rural population with PHC was moderate. Moreover, health facilities including UHFWC, long waiting time and even getting free medicine were negatively associated with PS. Findings suggest that policymakers should provide emphasis on improving service efficiency, medicine availability and patient‐centered care.

## 1 Introduction

Primary health care (PHC) has been regarded as essential for attaining the global goal of “health for all,” as established by the Alma‐Ata declaration in September 1978 [1]. PHC is a holistic strategy that addresses a diverse array of health concerns, such as disease prevention, diagnosis, treatment, and rehabilitation [2]. According to WHO, primary health care is a comprehensive societal strategy aimed at efficiently organizing and enhancing national health systems to deliver health and wellness services closer to communities. It is widely accepted as the most inclusive, equitable, and cost‐effective method to attain universal health coverage (UHC) [3]. It is also regarded as the primary line of defense against health issues [4]. PHC services are delivered by a collaborative team of healthcare professionals, including physicians, nurses, and midwives, who collectively treat patients' physical, emotional, and social requirements [5]. It emphasizes health education, counseling, and community participation in its goal of preventing disease and promoting health [2]. PHC is also regarded as essential for achieving the sustainable development goals (SDGs) [6]. This approach facilitates the attainment of good health at a low cost [7] by offering necessary and affordable health treatments, including health promotion, maternal, infant, and child healthcare, immunizations, and treatment for frequent illnesses throughout the lifespan [6]. PHC services are provided through a mixed public and private system serving a large rural population in Bangladesh [8]. The total health expenditure in 2020 was Taka 777 billion, which accounted for 2.8% of the gross domestic product (GDP), according to the Bangladesh National Health Accounts (BNHA) [9].

PHC functions as a consistent access point, facilitating close to health services, hence enhancing accessibility and mitigating social inequities. This will ultimately enhance patient satisfaction and result in improved compliance and efficient resource utilization [10]. The patient satisfaction (PS) refers to patients' evaluation of the healthcare services they received [6]. Patients are the ultimate users of primary healthcare, enabling them to locate areas for enhancement and meet user requirements, such as waiting times and staff communication [10]. Thus, the evaluation of patients' satisfaction with delivered PHC services is therefore crucial for assessing care quality and identifying areas for improvement and identifying possible areas for expanding the scope of primary healthcare practices [11].

In addition, there is a link between patient satisfaction and higher patient compliance, continuity of care, improved clinical services, increased service utilization, and effective risk management [12]. Insufficient patient satisfaction with medical services is a critical issue that contributes to the loss of doctor‐patient relationships, which in turn leads to a reduction in the utilization of medical services, particularly within primary care systems [13]. According to a recent study, the quality of healthcare provided by primary health care facilities in rural areas of China is inadequate, which may result in a drop in patient satisfaction [14]. However, another previous study based on primary health care facilities conducted in South Africa identified some utilizing factors that influenced patient satisfaction, such as distance to the clinic, number of visits, waiting times, effective care, and medicine availability [15]. Again, a study in India indicated that Patients reported satisfaction with primary healthcare, influencing factors like technical quality, communication, safety and cleanliness, and service costs [16]. Another recent study conducted in Nepal identified a positive correlation between responsiveness and perceived satisfaction in PHC settings, suggesting that patient satisfaction may be an important measure of service responsiveness. Patient satisfaction indicates that the healthcare system is adequately addressing their demands and responding efficiently [17]. Although Ali et al. reveals that high satisfaction with primary healthcare services in Pakistan, dissatisfaction is apparent due to low childbirth rates and the lack of ambulances for emergencies [18].

However, the primary healthcare system in Bangladesh is notably insufficient in accessibility and quality, especially in rural regions [4]. Adhikary et al. conducted a study in two divisions (Sylhet and Rajshahi) of Bangladesh found a considerable number of patients are dissatisfied with the care received [19]. Another study found only half of the total respondents utilized primary health care from the nearest community clinic [8]. Several studies on the utilization of primary healthcare services that were carried out in the northern regions of Bangladesh [8, 19, 20]. However, there is a scarcity of study conducted in the southern region of Bangladesh, which represents the coastal zone.

Additionally, it is essential to conduct study regarding the utilization factors that have an impact on the level of patient satisfaction in coastal regions, Bangladesh where Disasters, rapid population expansion, urbanization, and economic inequality have imposed significant pressure on the healthcare system, and there is an obvious absence of systematic evaluation of public opinion concerning the quality of health services [21]. Thus, the aim of the current study is to determine overall patient satisfaction in primary health care and to identify the association of patient satisfaction with sociodemographic variables and utilization characteristics among the rural community people in Khulna Division, Bangladesh. Patient satisfaction was determined using seven structured statements through a five‐point Likert scale. By identifying the factors associated with higher or lower satisfaction scores, this study intends to recognize areas for improvement to enhance the quality of primary healthcare services provided for the community people.

The outcome of this study provides insight into the patient satisfaction among users of primary health care services, which are vital for SDG Goal 3 to achieve UHC by 2030. Understanding factors like utilizing characteristics of PHC associated with satisfaction can help to highlight the necessity of assuring healthy lifestyles and well‐being for everyone, accessible through an effective primary health care system [21].

## 2 Methods

### 2.1 Study Design and Setting

The healthcare delivery system in Bangladesh comprises a complicated network of public health departments, non‐governmental organizations (NGOs), and private institutions [9]. The rural community can access healthcare services from several sources, including the government sector (community clinics, upazilla health complex (UHC), union health and family welfare centers (UHFWC), district hospitals, etc.), traditional medicine (Ayurvedic, Kabiraji, and Homeopathic), NGOs and other non‐profit entities, local pharmacies, and the private sector (such as private practitioners and clinics) [8, 19].

A community based cross‐sectional study was carried out from June to August 2024 in Khulna division of Bangladesh. Khulna division is located at southwestern part of Bangladesh on the banks of the Rupsha River and the Bhairab River consisting of 10 districts with the area of 22,285 square kilometers and a population of 15,563,000 [22]. Participants were selected using a multistage random sampling procedure. Three of the ten districts in the Khulna Division (Jashore, Kushtia, and Khulna) were randomly selected in the first stage. In the second stage, two sub‐districts were randomly selected from each district.

The main inclusion criteria of this study were rural people who were receiving health care services from the primary healthcare setting in the last 2 months, aged 18 or above. While inability to speak, and those rural people who were not received treatment from primary healthcare facility were excluded from the surveyed data.

### 2.2 Sample Size and Study Population

A minimum sample size of 384 was determined based on an unspecified prevalence of patient satisfaction (therefore assuming 50% prevalence) with a 5% permissible margin of error at the 95% confidence level and a 95% response rate. However, the sample size of the present study exceeded this. We interviewed 856 participants and asked for their participation in this study. Of the total, 135 did not meet the inclusion requirements resulting in 721 participants enrolled in the current study (final response rate 84.2%).

### 2.3 Questionnaire and Data Collection Techniques

A structured questionnaire was developed to collect information from individuals by a thorough review of the relevant literature [19, 20, 23]. Questionnaires were translated from English to Bengali (the local language) and subsequently back‐translated to English to ensure uniformity. The questionnaire was tested by conducting a pilot study among a small group of participants (*n* = 30) in order to further polish the language in the final version. The research team was advised by the results of the pilot study to modify certain sections of the questionnaire that the participants regarded as vague and unclear. Additionally, the results enabled us to enhance the methods or instruments. The questionnaires consisted of three parts including (i) socio‐demographic variables (ii) patient satisfaction related questions and lastly (iii) utilization of primary health care.

Data were collected through door‐to‐door visits using face‐to‐face interview from rural participants of the study location. Prior to data collection, a team of three research assistants received training on study objectives, ethical considerations, and standardized interviewing procedures. Participants were informed of the study's objective and consent was obtained from them. Respondents were assured that any information they gave would be kept confidential. The people who were being interviewed were told that they might withdraw at any time. Each interview lasted approximately 15–20 min and was held in a private space within the participant's residence to maintain confidentiality.

### 2.4 Outcome Variable

The outcome variable was patient satisfaction score. Overall patient satisfaction score was determined using structured questionnaire. Patient satisfaction questionnaire was consisted of seven questions including satisfaction with the amount charged for services, the convenient opening hour, the attitude of medical officer/medical staff/health care provider, the facility's environment, cleanliness of the facility, privacy settings of facility, and the supply of drugs. These questions were measured on a five‐point Likert scale defined as 0 = very dissatisfied, 1 = dissatisfied, 2 = average satisfied, 3 = satisfied, and 4 = very satisfied. Then, a satisfaction sum score for each participant was measured. This calculation produced an overall score, ranging from 0 to 28. The higher the score indicates the greater satisfaction of the participants. This questionnaire tool showed an acceptable reliability with a Cronbach's α of 0.73.

### 2.5 Independent Variables

Independent variables investigated in this study were socio‐demographic variables and utilization of primary health care. Socio‐demographic information including location (Jashore, Khulna, and Kushtia), age (18–30 years, 31–40 years, 41–50 years, > 50 years), gender (male or female), education level (illiterate, primary, secondary, and higher), marital status (married, unmarried, divorced, widowed), family member (≤ four members, more than four members), monthly family income (41.08–82.17 USD, 82.17–164.34 USD, 164.34–246.51 USD, > 246.51 USD), Chronic disease (no or yes). Health facilities include homeopathy, pharmacy, village doctor, union health and family welfare center (UHFWC), upazilla health complex (UHC), community clinic (CC). The organization of PHC facilities in Bangladesh is as follows: The upazilla health complex (UHC) is located at the upazilla level, which is a sub‐district that is a geographical region used for administrative purposes similar to that of a borough in Western countries. The union health and family welfare centers (UHFWC) exist at the union level, and community clinics (CC) are positioned at the community level [19]. Despite the fact that homeopathy, village doctor, and pharmacy are not technically included in the government PHC structure, they are frequently used in rural areas and were included to accurately reflect the primary health care patterns of the community. Chronic disease including diabetes, hypertension, breast cancer, heart disease, arthritis, chronic kidney disease, COPD, stroke, asthma, liver cirrhosis, chronic obesity, osteoporosis, hypothyroidism, mental and behavior disorder. Chronic disease condition was self‐reported collected from participants. Utilization characteristics of PHC including distance from home (< 3 km, ≥ 3 km), travel cost (0, < 0.16 USD, ≥ 0.16 USD), reasons for utilization (vicinity to home, low/free cost of treatment, low cost of transportation, quality of treatment, spouses/family decision), waiting time (0–15 min, 16–30 min, > 30 min), facing challenges (no difficulties, poor road conditions, long distance), feel comfortable asking question about health problems to the provider (no or yes), Number of care provider (one or more than one), Provision of prescription (not prescribed, prescribed), provision of free medicine (no or yes).

### 2.6 Statistical Analysis

The established guidelines outlined by Assel et al. [24] were followed to conduct data analysis and interpret the study's findings. All statistical analyses were performed using IBM SPSS Statistics (version 25.0) and R (version 4.3.0). At first, collected data were entered into SPSS and data were preprocessed. Then preprocessed data were converted into excel file and finally imported into an R studio for analysis. Simple descriptive analysis was done to observe frequency and percentage of socio‐demographic variables. Then t‐tests and one‐way ANOVA (Analysis of Variance) tests were performed to assess the association of the patient satisfaction scores with socio‐demographic variables and utilization characteristics. Finally, linear regression model was used to determine significant predictors of patient satisfaction. Multivariable linear regression analysis was performed with all theoretically relevant sociodemographic and healthcare utilization variables entered simultaneously into the model. This process maintained the retention of variables established in previous studies [8, 19], irrespective of their bivariate statistical significance. Potential confounding was mitigated by controlling all factors incorporated in the model. Cluster‐robust standard errors were computed at the district level using the CR2 variance estimator [25], which was applied through the lm_robust() function from the estimatr package in R, to address potential intra‐district correlation among observations. Prior to conduct the regression model, model diagnostic procedures were performed to evaluate the regression assumptions. The generalized variance inflation factor (GVIF) was employed to measure multicollinearity among the predictor variables in the multivariable model. We examined the GVIF values of each predictor, adjusting them for the degrees of freedom (df), to ensure that no variable significantly increased the variance of the regression coefficients. A threshold of GVIFˆ(1/(2*df)) ≤ 2 was used to indicate acceptable multicollinearity levels [26]. The GVIFˆ (1/(2*df)) of variables ranged 1.07–1.92, indicating no multicollinearity between the predictors. The residual normality was assessed visually using histograms and normal Q–Q plots, which revealed an approximately normal residual distribution with only minimal tail deviations (Supporting file). The Breusch–Pagan test was utilized to evaluate homoscedasticity. Despite the detection of heteroscedasticity, its possible effects were mitigated by employing district‐level cluster‐robust standard errors. The Cook's distance was employed to analyze influential observations, and the maximal Cook's distance was 0.036, suggesting that no highly influential observations were present. The statistical significance level was defined at a p‐value of less than 0.05. The reproducible table was produced by Gtsummary [27].

### 2.7 Ethics Approval and Consent to Participate

The ethical committee of the Faculty of Biological Science and Technology at Jashore University of Science and Technology, Jashore‐7408, Bangladesh, recognized and approved the study protocol (Ref: ERC/FBST/JUST/2024‐196). Informed consent, both written and verbal, was obtained prior to participation in the study. Data collection was performed in compliance with the ethical principles established in the Declaration of Helsinki. Keeping confidentiality in data collection, the data collector, supervisor, and investigator used code number instead of the Respondent's name. All participants provided written consents of their willingness to participate in the study.

## 3 Results

### 3.1 Socio‐Demographic Variables

Table 1 illustrates that a total of 721 respondents participated in the survey, with different location as 36.6%, 32.0%, and 31.3% from Jashore, Khulna and Kushtia, respectively. Age‐wise, 37.9% of respondents aged between 18 and 30 years and 14.4% aged more than 50 years. Furthermore, 61.2% being female, 20.7% illiterate and 36.8% had primary education. Among the respondents, housewife (51.6%), day laborer (15.4%), and married (84.6%). Consequently, 45.9% had income range between 82.17 to 164.34 USD and 54.9% had no chronic disease. Moreover, 49% respondents chose public healthcare access while 36.8% reported that they got primary healthcare from village doctor.

**Table 1:** Socio‐demographic characteristics of participants (*n* = 721).

Characteristic	*n* (%)
**Location**	
Jashore	264 (36.6)
Khulna	231 (32.1)
Kushtia	226 (31.3)
**Age**	
18–30	273 (37.9)
31–40	209 (29.0)
41–50	135 (18.7)
> 50	104 (14.4)
**Gender**	
Male	280 (38.8)
Female	441 (61.2)
**Education**	
Illiterate	149 (20.7)
Primary	265 (36.8)
Secondary	232 (32.2)
Higher	75 (10.4)
**Profession**	
Unemployed	27 (3.7)
Housewife	372 (51.6)
Day labor	111 (15.4)
Farmer	54 (7.5)
Service holder	25 (3.5)
Businessman	66 (9.2)
Student	66 (9.2)
**Marital status**	
Married	610 (84.6)
Unmarried	80 (11.1)
Divorced	5 (0.7)
Widowed	26 (3.6)
**Family member**	
< 4	473 (65.6)
≥ 4	248 (34.4)
**Monthly Family Income (USD)**	
41.08–82.17	228 (31.6)
82.17–164.34	331 (45.9)
164.34–246.51	115 (16.0)
> 246.51	47 (6.5)
**Chronic disease**	
No	396 (54.9)
Yes	325 (45.1)
**Health facility**	
Homeopathy	38 (5.3)
Pharmacy	58 (8.0)
Village doctor	265 (36.8)
Union health and family welfare center	73 (10.1)
Upazilla health complex	124 (17.2)
Community clinic	163 (22.6)

*Note:* 1 USD = 121.70 BDT.

### 3.2 Utilization Characteristics of PHC

Table 2 presents the healthcare utilization characteristics of the study participants (n = 721). Majority of the respondents (84%) resided within 3 km of the health facility, while only 16% traveled ≥ 3 km. Two‐thirds of the participants (65.9%) reported no travel cost, and only 10% incurred travel expenses ≥ 0.16 USD. Regarding reasons for utilizing the facility, the most frequently reported reason was the perceived quality of treatment (35.6%), followed by vicinity to home (28.4%) and low or free cost of treatment (22.7%). Most of the respondents (94%) reported facing no major challenges when utilizing health care. Moreover, nearly half of the participants (49%) experienced a waiting time of 0–15 min, while 23% waited 16–30 min, and 28% reported waiting more than half an hour. Most of the participants (87%) reported feeling comfortable asking questions about their health problems. Additionally, about 72.7% of participants attended to by a single care provider, while 27.3% consulted more than one provider during their visit. Approximately half of the participants (49%) received a prescription for medicine, whereas only 38.6% reported receiving free medicine.

**Table 2:** Utilization characteristics of PHC among the participants (*n* = 721).

Characteristic	*n* (%)
**Distance from home**	
< 3 km	606 (84)
≥ 3 km	115 (16)
**Travel costs (USD)**	
0	475 (65.9)
< 0.16	174 (24.1)
≥ 0.16	72 (10.0)
**Reason for utilization**	
Vicinity to home	205 (28.4)
Low/free cost of treatment	164 (22.7)
Low cost of transportation	7 (1.0)
Quality of treatment	257 (35.6)
Spouses/family decision	88 (12.2)
**Facing challenge**	
No difficulties	678 (94)
Poor road condition	12 (1.7)
Long distance	31 (4.3)
**Waiting time**	
0–15 min	353 (49)
16–30 min	167 (23)
> 30 min	201 (28)
**Feel comfortable asking questions about health problems to the provider**	
No	96 (13.3)
Yes	625 (86.7)
**Number of care provider**	
One	524 (72.7)
More than one	197 (27.3)
**Provision of prescription**	
Not prescribed	368 (51)
Prescribed	353 (49)
**Free medicine**	
No	443 (61.4)
Yes	278 (38.6)

Table 3 outlines the responses of participants on patient satisfaction statements. Based on the seven items, the highest percentage of patient satisfaction was observed in the environment of the facility. Specifically, 79.2% of participants reported being satisfied and 7.6% were very satisfied with the facility environment. Similarly, satisfaction with cleanliness of the facility was notably high, with 77.1% satisfied and 8% very satisfied. These two items claimed the highest mean scores (2.89 ± 0.61 and 2.87 ± 0.65, respectively), suggesting positive perceptions regarding infrastructure and hygiene conditions. A large proportion of patients also expressed satisfaction with financial and service accessibility items. Approximately 74.1% were satisfied with the amount charged for services, and 72.5% were satisfied with convenient opening hours. Satisfaction with the attitude of medical officer or staff or health care provider was also high, with 69.3% reporting satisfaction and 8% very satisfied (mean = 2.74 ± 0.77). In contrast, comparatively lower satisfaction levels were observed in privacy and drug supply. Only 54.8% of respondents were satisfied with the privacy settings of the facility, while 16.8% reported overall dissatisfaction. The mean score for privacy (2.46 ± 0.96) was lower than most other domains. The lowest satisfaction was reported for the supply of drugs. Notably, 44.3% of participants expressed overall dissatisfaction and only 1.7% were very satisfied. This item recorded the lowest mean score (1.83 ± 1.07). Additionally, the overall PS score was 18.41 ± 3.49 (range: 0–28). This reflects an overall satisfaction rate of 65.8% (18.41/28 × 100), indicating a moderate level of satisfaction.

**Table 3:** Descriptive statistics of patient satisfaction among the participants (*n* = 721).

Statements	5‐Point likert scale of patient satisfaction, *n* (%)					Satisfaction score
	Very dissatisfied	Dissatisfied	Average satisfied	Satisfied	Very satisfied	Mean ± SD
Satisfaction with the amount charged for services	5 (0.7)	50 (6.9)	69 (9.6)	534 (74.1)	63 (8.7)	2.83 ± 0.70
Satisfaction with the convenient opening hour	24 (3.3)	45 (6.2)	83 (11.5)	523 (72.5)	46 (6.4)	2.72 ± 0.81
Satisfaction with the attitude of medical officer/medical staff/health care provider	6 (0.8)	69 (9.6)	88 (12.2)	500 (69.3)	58 (8)	2.74 ± 0.77
Satisfaction with the environment of the facility	8 (1.1)	22 (3.1)	65 (9.0)	571 (79.2)	55 (7.6)	2.89 ± 0.61
Satisfaction with the cleanliness of the facility	9 (1.2)	27 (3.7)	71 (9.8)	556 (77.1)	58 (8)	2.87 ± 0.65
Satisfaction with the privacy settings of facility	36 (5)	85 (11.8)	156 (21.6)	395 (54.8)	49 (6.8)	2.46 ± 0.96
Satisfaction with the supply of drugs	79 (11)	240 (33.3)	141 (19.6)	249 (34.5)	12 (1.7)	1.83 ± 1.07
**Overall satisfaction score**	NA	NA	NA	NA	NA	18.41 ± 3.49

Abbreviations: NA, not applicable.

Table 4 presents the association between socio‐demographic and utilization characteristics and patient satisfaction score among the respondents (*n* = 721). Patient satisfaction scores differed significantly by socio‐demographic variables including gender (*t* = −2.099, *p* = 0.036), monthly family income (*F* = 3.298, *p* = 0.020), chronic disease (*t* = 3.637, *p* < 0.001), health facility (*F* = 27.261, *p* < 0.001). Regarding utilization characteristics, reasons for utilization (*F* = 22.809, *p* < 0.001), waiting time (*F* = 41.611, *p* < 0.001), feel comfortable asking question about health problems to the provider (*t* = −10.530, *p* < 0.001) and free medicine (*t* = 9.271, *p* < 0.001) were significantly associated with patient satisfaction scores.

**Table 4:** Association between socio‐demographic and utilization characteristics and patient satisfaction score among the participants (*n* = 721).

	Patient satisfaction score		
Variables	Mean ± SD	F/t‐test	*p* value
**Gender**		−2.099	**0.036**
Male	18.07 ± 3.17		
Female	18.63 ± 3.66		
**Age (years)**		1.551	0.200
18–30	18.65 ± 3.70		
31–40	18.54 ± 3.04		
41–50	17.94 ± 3.61		
> 50	18.14 ± 3.59		
**Education**		0.652	0.582
Illiterate	18.36 ± 3.44		
Primary	18.31 ± 3.45		
Secondary	18.66 ± 3.27		
Higher	18.12 ± 4.35		
**Profession**		1.536	0.164
Unemployed	17.85 ± 3.44		
Housewife	18.76 ± 3.56		
Day labor	17.83 ± 3.11		
Farmer	18.24 ± 3.57		
Service holder	18.68 ± 3.35		
Businessman	18.19 ± 2.96		
Student	17.94 ± 4.07		
**Marital status**		1.934	0.161
Married	18.45 ± 3.48		
Unmarried	17.88 ± 3.94		
Divorced	20.00 ± 2.00		
Widowed	19.00 ± 2.23		
**Family member**		1.156	0.248
< 4	18.52 ± 3.62		
≥ 4	18.21 ± 3.23		
**Monthly Family Income (USD)**		3.298	**0.020**
41.08–82.17	18.68 ± 3.62		
82.17–164.34	18.29 ± 3.23		
164.34–246.51	17.81 ± 3.87		
> 246.51	19.51 ± 3.41		
**Chronic disease**		3.637	**< 0.001**
No	18.84 ± 3.37		
Yes	17.89 ± 3.57		
**Health facility**		27.261	**< 0.001**
Homeopathy	20.55 ± 2.31		
Pharmacy	19.72 ± 1.82		
Village doctor	19.67 ± 2.45		
Union health and family welfare center	16.64 ± 3.85		
Upazilla health complex	17.11 ± 3.65		
Community clinic	17.18 ± 4.09		
**Distance from home**		1.044	0.216
< 3 km	18.48 ± 3.32		
≥ 3 km	18.04 ± 4.28		
**Travel costs (USD)**		1.114	0.331
0	18.53 ± 3.39		
< 0.16	18.36 ± 3.33		
≥ 0.16	17.75 ± 4.37		
**Reason for utilization**		22.809	**< 0.001**
Vicinity to home	17.95 ± 3.43		
Low/free cost of treatment	16.65 ± 3.86		
Low cost of transportation	16.86 ± 5.33		
Quality of treatment	19.69 ± 2.61		
Spouses/family decision	19.15 ± 3.39		
**Facing challenge**		2.135	0.142
No difficulties	18.51 ± 3.38		
Poor road condition	16.83 ± 4.80		
Long distance	16.97 ± 4.89		
**Waiting time**		41.611	**< 0.001**
0–15 min	19.38 ± 2.68		
16–30 min	18.88 ± 2.81		
> 30 min	16.32 ± 4.29		
**Feel comfortable asking questions about health problems to the provider**		−10.530	**< 0.001**
No	14.44 ± 4.09		
Yes	19.02 ± 2.95		
**Number of care provider**		1.399	0.162
One	18.52 ± 3.48		
More than one	18.11 ± 3.51		
**Provision of prescription**		0.126	0.900
Not prescribed	18.43 ± 3.53		
Prescribed	18.39 ± 3.46		
**Free medicine**		9.271	**< 0.001**
No	19.39 ± 2.66		
Yes	16.85 ± 4.05		

### 3.3 Factors Associated With Patient Satisfaction (PS)

The linear regression model in Table 5 revealed that participants aged 41–50 years (*β* = −0.69; *p* = 0.034) had significantly lower PS score, as compared with reference age category. Respondents who had chronic disease (*β* = −0.77; *p* = 0.047) had lower PS score compared to those who had no chronic disease. Moreover, the PS score was also significantly lower in union health and family welfare center (*β* = −1.7; *p* = 0.028) as compared to homeopathy. Regarding utilization characteristics, quality of treatment (*β* = 1.5; *p* = 0.036) was a reason for using PHC that was a significant predictor of having higher PS score as compared to their reference reason category. Waiting time for more than a half hour (*β* = −1.7; *p* = 0.023) was another significant predictor of lowering PS score as compared to their reference category. Participants who could ask questions to the care provider comfortably (*β* = 2.8; *p* = 0.038) had greater satisfaction score than who could not ask in comfortable way. However, participants who got free medicine (*β* = −1.7; *p* = 0.032) had less satisfaction score as compared with the participants who got free medicine. The model explained 41.68% of the variance in the outcome (*R* ^2^ = 0.4168; adjusted *R* ^2^ = 0.3816).

**Table 5:** Linear regression analysis of factors associated with patient satisfaction among the participants (*N* = 721).

Characteristic	*β*	95% CI	*p* value
**Gender**			
Male	RC	—	
Female	0.43	−3.5, 4.4	0.687
**Age (years)**			
18–30	RC	—	
31–40	−0.53	−1.1, 0.01	0.052
41–50	−0.69	−1.2, −0.13	**0.034**
> 50	−0.94	−2.3, 0.37	0.090
**Education**			
Illiterate	RC	—	
Primary	−0.11	−2.0, 1.8	0.832
Secondary	−0.25	−2.0, 1.5	0.601
Higher	−0.66	−2.4, 1.1	0.245
**Profession**			
Unemployed	RC	—	
Housewife	−0.37	−7.4, 6.7	0.833
Day labor	−0.30	−5.5, 4.9	0.818
Farmer	0.01	−5.8, 5.8	0.996
Service holder	−0.44	−4.4, 3.5	0.655
Businessman	−0.86	−3.9, 2.2	0.331
Student	−0.74	−7.7, 6.2	0.675
**Marital status**			
Married	RC	—	
Unmarried	−1.0	−3.1, 0.98	0.153
Divorced	1.1	−1.9, 4.1	0.237
Widowed	−0.13	−2.3, 2.0	0.787
**Family member**			
< 4	RC	—	
≥ 4	−0.08	−1.6, 1.4	0.835
**Monthly Family Income (USD)**			
41.08–82.17	RC	—	
82.17–164.34	−0.07	−2.0, 1.9	0.887
164.34–246.51	−0.62	−2.5, 1.2	0.289
> 246.51	0.27	−1.8, 2.3	0.603
**Chronic disease**			
No	RC	—	
Yes	−0.77	−1.5, −0.03	**0.047**
**Health facility**			
Homeopathy	RC	—	
Pharmacy	−0.70	−5.0, 3.6	0.552
Village doctor	−1.2	−3.5, 1.1	0.142
Union health and family welfare center	−1.7	−3.0, −0.46	**0.028**
Upazilla health complex	−0.60	−1.6, 0.39	0.118
Community clinic	−1.7	−5.2, 1.8	0.165
**Distance from home**			
< 3 km	RC	—	
≥ 3 km	−0.02	−1.6, 1.6	0.954
**Travel costs (USD)**			
0	RC	—	
< 0.16	−0.32	−2.1, 1.4	0.516
≥ 0.16	0.14	−3.1, 3.4	0.863
**Reason for utilization**			
Vicinity to home	RC	—	
Low/free cost of treatment	−0.01	−1.1, 1.0	0.962
Low cost of transportation	−2.2	−11, 6.3	0.383
Quality of treatment	1.5	0.23, 2.7	**0.036**
Spouses/family decision	0.04	−0.54, 0.63	0.781
**Facing challenge**			
No difficulties	RC	—	
Poor road condition	−1.9	−11, 6.8	0.424
Long distance	−1.8	−10, 6.5	0.337
**Waiting time**			
0–15 min	RC	—	
16–30 min	−0.26	−0.92, 0.40	0.231
> 30 min	−1.7	−2.8, −0.59	**0.023**
**Feel comfortable asking questions about health problems to the provider**			
No	RC		
Yes	2.8	0.39, 5.2	**0.038**
**Number of care provider**			
One	RC	—	
More than one	0.33	−1.9, 2.6	0.591
**Provision of prescription**			
Not prescribed	RC	—	
Prescribed	0.03	−0.44, 0.51	0.805
**Free medicine**			
No	RC	—	
Yes	−1.7	−3.0, −0.35	**0.032**

*Note:* Bold indicates statistically significant value (*p* < 0.05). Standard errors were clustered at the district level to cope with potential intra‐district correlation among observations. *R* ^2^ = 0.4168, adjusted *R* ^2^ = 0.3816

Abbreviations: *β*, regression coefficient; CI, confidence interval; RC, reference category.

## 4 Discussion

Present study identified association of patient satisfaction with socio‐demographic variables and utilization characteristics among study participants residing in the Khulna division. The overall patient satisfaction score was 18.41 ± 3.49. In multivariable linear regression analysis, the study indicated some sociodemographic variables like participants aged 41–50 years, those with chronic diseases and health facility such as union health and family welfare center were significantly associated with lower patient satisfaction scores. Regarding utilization characteristics, having quality of treatment and getting comfortability to ask health provider were significantly associated with higher patient satisfaction score. However, extended waiting time (> 30 min) and getting free medicine were significant predictors of decreased patient satisfaction score.

In the current study, patient satisfaction average score of 18.41, representing 65.8% of the total score. However, a recent study conducted [11] in Saudi Arabia showed higher patient satisfaction (83.8% of the total score) which was greater than in our study. However, another recent study in Sierra Leone [28] found overall 63.8% satisfaction level among patient in five PHC facilities which is slightly lower than in our study. The differences in patient satisfaction might result from disparities in primary health care structure, patient expectations, and instruments for measurement. Present study revealed some reasons which were responsible for the satisfaction including service charge, convenient opening hours, attitude of healthcare provider, cleanliness, and environment of the facilities, privacy of the facilities and drug supply. On the other hand, Alhajri et al. [11] indicated that the main factor of patient satisfaction was credited to the care provider, followed by personal concerns (patient privacy, clinic cleanliness), and subsequently the nursing domain. Additionally, we found important utilizing factors along with some sociodemographic variables were associated with patient satisfaction level within study population in our study.

First, the survey results showed patient satisfaction linked with age, with participants aged 41 to 50 years tend to have lower satisfaction scores than their counterpart young participants. A study conducted in Brazil showed that elderly people exhibited dissatisfaction in primary healthcare [29]. Possible reason might be due to noncompliance with scheduled medical consultation dates, challenges in performing required diagnostics or obtaining specialist consultations, accessibility issues, and insufficient resources to provide healthcare to the older people [29]. We found lower patient satisfaction among chronic patients as compared to non‐chronic patients which was contrast. This result was found contradict with recent previous study conducted in South Africa which reported high satisfaction level among chronic patient [15]. There are certain reasons responsible for this discrepancy. All tiers of primary health care are presently unprepared for dealing with chronic diseases in Bangladesh. One of the most prominent issues was the lack of trained workers and guidelines, as well as resources for diagnosis and necessary medicine [30]. According to World Bank, health workers within Bangladesh's primary health‐care system have not yet received training in the treatment of non‐communicable diseases (NCDs) [31]. In terms of health facilities, patient satisfaction was lower in UHFWC as compared to homeopathy setting. A recent study conducted in Iraq identified some important reasons which were responsible for dissatisfaction among patients including unavailability of essential drugs, lack of health education programs, long waiting time, lack of medical instruments in primary health care setting [32].

We also found that utilizing factors of primary health care influencing patient satisfaction in the study population through regression analysis. A noteworthy finding is that when patients get quality of treatment, it increased satisfaction scores. This finding is in line with past study stated that people were satisfied and comfortable in the services provided by healthcare providers [11]. This demonstrates that efficient treatment and collaboration with health care providers are essential for increasing satisfaction level among patients. In the study, satisfaction score decreased with increasing waiting time of more than 30 min as compared to wait less than 15 min. This result was consistent with a prior study that showed around 25% percent of patients had to wait more than 30 min to see their primary care doctor in a clinic and the study showed that general satisfaction goes down as waiting time goes up [16]. However, the Institute of Medicine reports that a minimum of 90% of patients ought to receive medical care within 30 min of their planned appointment. Prolonged waiting times can negatively impact the utilization of health services, continuity of care, and patient satisfaction [33]. Another worth finding of our study is that patient satisfaction was significantly associated with feeling comfortable to ask health related problems to the healthcare provider. A recent study conducted in rural China based on primary health facilities also indicated that patient satisfaction is greatly influenced by the physician's capacity to promote a feeling of relaxation in patients, encouraging them to share their symptoms and worries [13]. In addition, regression result indicated dissatisfaction was unexpectedly found among patients who received free medicines. A reasonable justification may be shortages of medicines, primarily resulting from institutions' deficiencies in essential drugs [34]. This indicates that access to essential medicines may be restricted, suggesting that free medicine is not reliably accessible.

The findings of the current study should, however, be interpreted with caution. Some observed associations particularly perceived quality of treatment and felt comfortable asking questions about health problems to the provider are closely linked to the patient experience and may conceptually overlap with the satisfaction outcome. Therefore, these observed associations may partially reflect measurement overlap rather than independent determinants of satisfaction.

### 4.1 Policy Implications

Patients usually seek timely, readily accessible, and quality healthcare services when utilizing primary health care [28]. Although this study found a moderate level of overall satisfaction among rural populations, several service‐related and utilization factors were identified that require attention to improve experiences within the primary healthcare system. The findings indicate the comparatively low satisfaction score related to drug supply highlights the need to ensure consistent availability of essential medicines in primary healthcare facilities, which remains a critical component of quality service delivery. This study also identified waiting time showed a particularly strong association with this finding underscores the importance of improving patient flow management and optimizing service organization to minimize delays and enhance service efficiency in primary healthcare facilities. Another notable finding is the strong relationship between patient satisfaction and communication with care providers. This emphasizes the importance of strengthening patient‐centered care practices, including respectful communication, active listening and clear explanations from healthcare providers. Furthermore, integrating routine patient satisfaction monitoring and incorporating community feedback into service planning may support healthcare managers and policymakers in improving the responsiveness and quality of primary healthcare services. Improved health outcomes and UHC need building trust in the PHC system through timely and efficient service delivery.

### 4.2 Strengths and Limitations

This study possesses various strengths. The diverse and large sample size, comprising individuals from different socioeconomic backgrounds, both genders, and numerous geographic areas, improves the generalizability of the findings. The implementation of a cross‐sectional design provides an overview of patient satisfaction regarding primary health care, enhancing comprehension of the present state of the utilization of primary health care services.

However, there are still some limitations to this study. The cross‐sectional nature implies that only relationships can be drawn, and causality cannot be established. Moreover, the patient satisfaction surveys were administered within 2 months after the patient's last primary health care visit, which would have caused recall bias. Additionally, the findings are based on respondents who had used PHC services within the last 2 months. Therefore, the results may not be fully generalizable to all rural residents, particularly individuals who were non‐users of PHC services. Some utilization characteristics, such as “quality of treatment” (included under “reason for utilization” variable) and “feel comfortable asking questions about health problems to the provider” may be conceptually linked to elements of patient experience. However, PS was assessed using a distinct multi‐item scale covering different aspects of patient experience. Hence, these variables were regarded as contextual factors influencing satisfaction rather than elements of the outcome itself.

## 5 Conclusion

The overall patient satisfaction with primary health care was moderate among the rural community people in Khulna division. Our findings suggest that the socio‐demographic characteristics and utilization factors are associated significantly with the satisfaction. To be more precise, the patients who aged 41 to 50, had chronic disease, and received healthcare from UHFWC were associated negatively with satisfaction. Respondents who received a good quality of treatment, and comfort to ask questions were positively associated with satisfaction. However, long waiting time and even getting free medicine from the facility were linked with lower satisfaction. The findings will help policymakers and stakeholders identify needs of improving patient satisfaction and develop comprehensive multisectoral strategies to meet the requirements for achieving maximum patient satisfaction. It will also contribute to increasing trust among people about primary health care sector which has positive impact on overall health sector.

## Author Contributions

**Md. Moshiuzzaman Adnan:** data curation, formal analysis, writing – original draft, writing – review and editing. **Afsana Tanni:** data curation, writing – original draft, writing – review and editing. **Israt Jahan:** data curation, writing – original draft, writing – review and editing. **Suvasish Das Shuvo:** conceptualization, methodology, writing – original draft, writing – review and editing; validation.

## Funding

The authors have nothing to report.

## Ethics Statement

The ethical committee of the Faculty of Biological Science and Technology at Jashore University of Science and Technology, Jashore‐7408, Bangladesh, recognized and approved the study protocol (Ref: ERC/FBST/JUST/2024‐196). Informed consent, both written and verbal, was obtained prior to participation in the study. Data collection was performed in compliance with the ethical principles established in the Declaration of Helsinki.

## Conflicts of Interest

The authors declare no conflicts of interest.

## Transparency Statement

All authors affirms that this manuscript is an honest, accurate, and transparent account of the study being reported; that no important aspects of the study have been omitted; and that any discrepancies from the study as planned (and, if relevant, registered) have been explained.

## Supporting information

Supporting File

## Data Availability

The data that support the findings of this study are available on request from the corresponding author. The data are not publicly available due to privacy or ethical restrictions.
