# Supplementary material for: Determinants of Patient Satisfaction With Primary Health Care Among the Rural Community People: A Cross‐Sectional Evidence From Khulna Division, Bangladesh
Source: Health Sci Rep. 2026 Aug 18;9(8):e73043. doi: 10.1002/hsr2.73043 (PMC13482351; doi:10.1002/hsr2.73043)
Supplement: Supplementary file 1 [file HSR2-9-e73043-s001.docx]

**Supplementary figures**

**Figure S1. Histogram of multivariable linear regression residuals (N = 721)**

**Figure S2 Normal Q–Q plot of multivariable linear regression residuals (N = 721)**
